# Oestrogen Inhibits Arterial Calcification by Promoting Autophagy

**DOI:** 10.1038/s41598-017-03801-x

**Published:** 2017-06-14

**Authors:** Yi-Qun Peng, Dan Xiong, Xiao Lin, Rong-Rong Cui, Feng Xu, Jia-Yu Zhong, Ting Zhu, Feng Wu, Min-Zhi Mao, Xiao-Bo Liao, Ling-Qing Yuan

**Affiliations:** 1 Department of Metabolism and Endocrinology, National Clinical Research Center for Metabolic Diseases, The Second Xiang-Ya Hospital, Central South University, Changsha, Hunan People’s Republic of China; 2 Department of Endocrinology, Central hospital of Yiyang, Yiyuang, Hunan People’s Republic of China; 3 Department of Pathology, The Second Xiang-Ya Hospital, Central South University, Changsha, Hunan People’s Republic of China; 4 Department of Orthopaedics, The Second Xiang-Ya Hospital, Central South University, Changsha, Hunan People’s Republic of China; 5 Departments of Cardiothoracic Surgery, The Second Xiang-Ya Hospital, Central South University, Changsha, Hunan People’s Republic of China

## Abstract

Arterial calcification is a major complication of cardiovascular disease. Oestrogen replacement therapy in postmenopausal women is associated with lower levels of coronary artery calcification, but its mechanism of action remains unclear. Here, we show that oestrogen inhibits the osteoblastic differentiation of vascular smooth muscle cells (VSMCs) *in vitro* and arterial calcification *in vivo* by promoting autophagy. Through electron microscopy, GFP–LC3 redistribution, and immunofluorescence analyses as well as measurement of the expression of the autophagosome marker light-chain I/II (LC3I/II) and autophagy protein 5 (Atg5), we show that autophagy is increased in VSMCs by oestrogen *in vitro* and *in vivo*. The inhibitory effect of oestrogen on arterial calcification was counteracted by 3-methyladenine (3MA) or knockdown of Atg5 and was increased by rapamycin. Furthermore, the inhibitory effect of oestrogen on arterial calcification and the degree of autophagy induced by oestrogen were blocked by a nonselective oestrogen receptor (ER) antagonist (ICI 182780), a selective oestrogen receptor alpha (ERα) antagonist (MPP), and ERα-specific siRNA. Our data indicate that oestrogen inhibits the osteoblastic differentiation of VSMCs by promoting autophagy through the ERα signalling pathway *in vitro* and arterial calcification *in vivo* by increasing autophagy. Our findings provide new insights into the mechanism by which oestrogen contributes to vascular calcification *in vitro* and *in vivo*.

## Introduction

Arterial calcification, a major complication of cardiovascular disease, is often found in patients with atherosclerosis, diabetes, renal failure, postmenopausal syndrome or aortic stenosis^1, 2^. Previously, arterial calcification was regarded as a passive consequence of ageing, renal failure and diabetes^1^. However, multiple lines of evidence have shown that vascular calcification resembles osteogenesis, and factors regulating bone mineralization have been demonstrated in calcified plaques^3–7^. The process of calcification requires the expression of several osteoblast phenotype genes, such as alkaline phosphatase (ALP), core binding factor α1 (Cbfα1 or Runx2), osteocalcin and osteopontin^5, 7^. Previous studies have demonstrated that vascular smooth muscle cells (VSMCs) play a pivotal role in the active regulation of vascular calcification by acquiring the phenotype of osteoblast-like cells^3–10^.

Autophagy is a highly conserved cellular process responsible for the removal or recycling of long-lived proteins and organelles and can provide cells with an alternative source of nutrients from the reuse of cellular proteins and organelles^11–15^. Autophagy plays an important role in cell growth, survival, differentiation, and homeostasis and in multiple diseases, such as neurodegenerative disease, cancer, heart disease and arteriosclerosis^13^. To some extent, autophagy can prevent the activation of apoptotic pathways through the removal of damaged mitochondria^16^. In some systems, autophagy can enhance the apoptotic response^17^. Multiple studies have reported that autophagy occurs in the context of atherosclerosis and hypertension^18–20^. There are evidences indicating that rapamycin-based drugs, which are inducers of autophagy, can prevent phenotype switching and hyperproliferation of VSMCs^21–23^. A recent study has shown that in the context of hyperphosphatemia, vascular calcification occurs and autophagy increases; thus, autophagy may function as an endogenous protective mechanism in attenuating the calcification of VSMCs^24^. These phenomena suggest that autophagy plays a pivotal role in arterial calcification.

Arterial calcification is often associated with osteoporosis. Thus, its incidence is much lower in premenopausal women. The role of sex hormones, especially oestrogen (E2), may explain the age discrepancy in susceptibility to arterial calcification. In the Women’s Health Initiative clinical trial, postmenopausal women treated with long-term oestrogen therapy had lower levels of coronary artery calcification^25^. Oestrogen can inhibit vascular lesion progression through anti-inflammatory responses^26^. However, the effect of oestrogen on arterial calcification and the mechanism involved have not been fully clarified.

A previous study demonstrated that autophagy induced by oestrogen exerts protective effects in the pathogenesis of hypoxia-induced pulmonary hypertension^27^. Here, we investigate whether oestrogen attenuates arterial calcification by regulating autophagy in VSMCs *in vitro* and *in vivo* and explore the mechanism involved.

## Results

### Autophagy is increased during the osteoblastic differentiation of VSMCs and in calcified arteries

**Figure 1:**
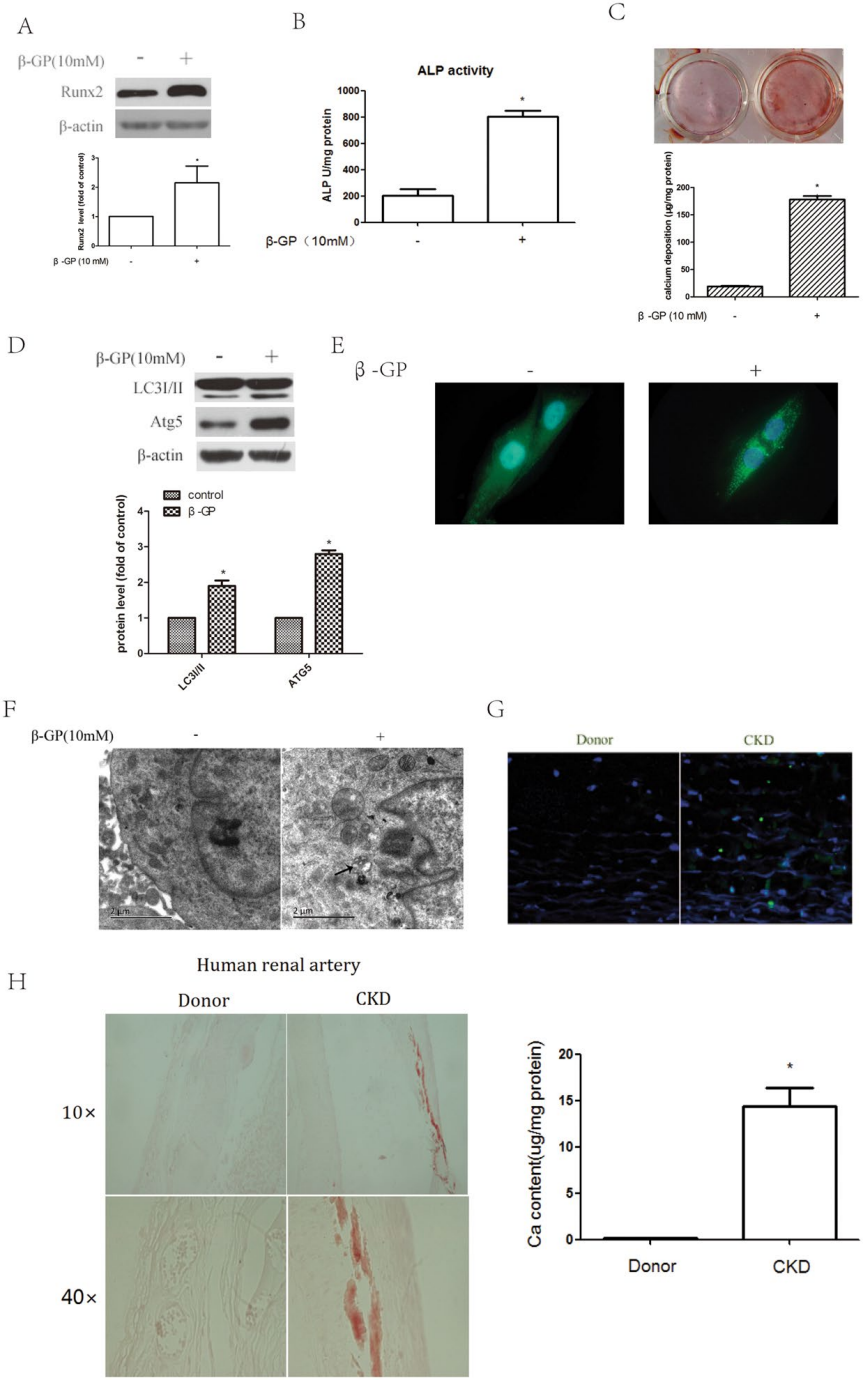
Autophagy is increased during the calcification of VSMCs and in calcified arteries. (**A**) Western blot analysis of Runx2 levels in VSMCs treated with 10 mM β-GP for 72 h. (**B**) Effect of β-GP on ALP activity. VSMCs were cultured with β-GP for 72 h; ALP activity was measured using an ALP kit. *p < 0.01 compared with the cells treated with vehicle (n = 5). (**C**) Effect of β-GP on calcium deposition. VSMCs were treated with β-GP for 12 days. A representative plate view of the Alizarin Red S staining is shown. Quantification of calcium levels using the O-cresolphthalein complexone method. *p < 0.01 compared with the cells treated with vehicle (n = 5). (**D**) Western blot analysis of LC3I/II and Atg5 levels in VSMCs treated with 10 mM β-GP for 72 h (n = 5). (**E**) Confocal microscopy of green fluorescent proteins (GFP) in VSMCs transiently transfected with GFP-LC3 plasmids cultured for 48 h and then treated with β-GP for 72 h. Autophagosomes are indicated by the fluorescent puncta. (**F**) VSMCs were incubated with β-GP for 72 h and then analysed by electron microscopy. A representative image is shown. Autophagosomal vacuoles containing organelle remnants are highlighted by arrows (n = 5). (**G**) Immunofluorescence analysis of LC3 puncta in the renal arterial wall from human organ donors and uremic patients during renal transplantation (n = 10). (**H**) Alizarin Red S staining was used to evaluate vascular calcification in the renal arterial wall from human organ donors and uremic patients (n = 10). (**I**) Calcium deposition in the arteries from human organ donors and uremic patients. Calcium was extracted with HCl and quantified by spectrophotometry using the O-cresolphthalein complexone method. Calcium content was measured by spectrophotometry using the O-cresolphthalein complexone method. Representative images are shown (n = 10). The data are expressed as the mean ± SD. *p < 0.01 compared with the donor group.

It is widely accepted that the process of vascular calcification is similar to that of bone mineralization. ALP, Runx2 and mineralized matrix are well-established phenotypic markers of osteoblasts and are upregulated during the osteoblastic differentiation of VSMCs^28, 29^. Our data showed that treatment with β-GP increases Runx2 expression (Fig. 1A) and ALP activity (Fig. 1B) in VSMCs. We used Alizarin Red S staining to determine matrix mineralization and found that β-GP enhanced Alizarin Red S staining and calcium deposition (Fig. 1C) in VSMCs, which was consistent with our previous findings that β-GP can induce the osteoblastic differentiation of VSMCs^4, 9, 10^.

To investigate whether autophagy is involved in the osteoblastic differentiation of VSMCs, we exposed VSMCs to β-GP and examined the effect of β-GP on autophagosome formation. We used three methods to identify autophagosome formation in our experiments. First, the expression of LC3I/II and Atg5 were detected by Western blotting. Conversion of the lipid-conjugated form of the autophagosome marker light-chain LC3-I into LC3-II is an essential step in autophagosome formation, and the abundance of LC3-II is correlated with the number of autophagosomes^30^. Atg5 also plays an important role in the initiation and elongation of autophagosomes. Therefore, LC3I/II and Atg5 can be used as markers of autophagy^31^. We found that β-GP treatment induced a marked increase in LC3I/II and Atg5 expression in VSMCs compared with that in the VSMCs treated with vehicle (Fig. 1D). Second, under basal conditions, LC3 is a diffuse cytosolic protein. After induction with β-GP, LC3 is proteolytically cleaved, lipidated and localized to autophagosomal membranes, forming punctate subcellular structures^30^. Treatment with β-GP led to a significant induction of autophagy as represented by the increased accumulation of LC3 puncta tagged with green fluorescent protein (GFP) (Fig. 1E). Third, electron microscopy of typical autophagic structures can provide direct evidence of autophagy activation. The high degree of autophagic activity in VSMCs was further confirmed at the ultrastructural level by electron microscopy, which demonstrated the accumulation of typical autophagic structures in cells exposed to β-GP (Fig. 1F). These results suggested that β-GP could stimulate autophagy in VSMCs, accompanied by the osteoblastic differentiation of VSMCs. To confirm that autophagy was involved in arterial calcification *in vivo*, we performed immunofluorescence analysis of LC3 puncta. We found that autophagosomes were formed in the renal arteries of uremic patients, whereas the renal arteries of donors showed no evidence of LC3 puncta (Fig. 1G). The renal arteries of uremic patients were calcified, which was confirmed by Alizarin Red S staining and calcium content (Fig. 1H). The clinical characteristics of the uremic patients and health donors are summarized in Supplementary Table 1. These results demonstrate the increase in autophagy in association with the osteoblastic differentiation of VSMCs and calcified arteries.

### Autophagy plays a protective role through the inhibition of osteoblastic differentiation of VSMCs

**Figure 2:**
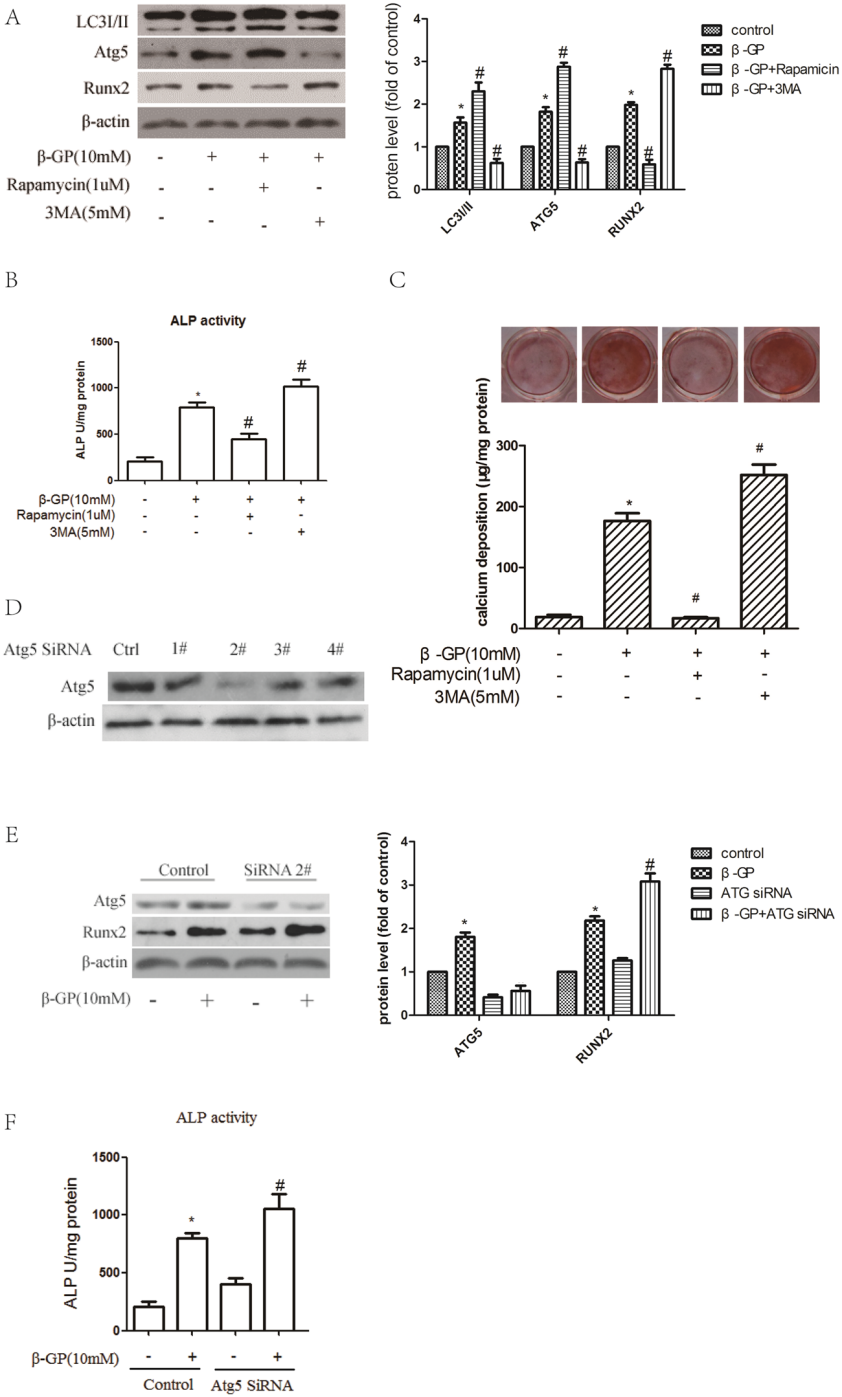
Autophagy plays a protective role in inhibiting the calcification of VSMCs. (**A**,**B**) VSMCs were pre-treated with the indicated concentrations of 3MA or rapamycin (3MA, an inhibitor of autophagy; rapamycin, an inducer of autophagy) for 30 min and were subsequently incubated with β-GP for 72 h. LC3I/II, Atg5 and Runx2 levels (**A**) and ALP activity (**B**) were detected using Western blot analysis or an ALP kit, respectively. *p < 0.01 compared with the cells treated with vehicle. ^#^ *p* < 0.01 compared with the cells treated with β-GP (n = 5). (**C**) VSMCs were pre-treated with the indicated concentrations of 3MA or rapamycin for 30 min and then incubated with β-GP for 12 days. A representative plate view of the Alizarin Red S staining is shown. Quantification of calcium levels using the O-cresolphthalein complexone method. *p < 0.01 compared with the cells treated with vehicle. ^#^ *p* < 0.01 compared with the cells treated with β-GP (n = 5). (**D**) Western blot analysis of Atg5 protein levels in VSMCs transfected with siRNA against Atg5 (Atg5 1#, Atg5 2#, Atg5 3# and Atg5 4#) or scrambled control for 48 h. (**E**,**F**) VSMCs were transfected with control or Atg5 2# siRNA for 48 h and treated with or without β-GP for 72 h. Western blot analysis of Atg5 and Runx2 protein levels (**E**) and ALP activity detection (**F**) were performed. *p < 0.01 compared with the cells treated with vehicle in the control group. ^#^ *p* < 0.01 compared with the cells treated with vehicle in the ATG5 siRNA group (n = 5). Representative images are shown.

To address the potential role of autophagy in the osteoblastic differentiation of VSMCs, rapamycin (1 µM), a pharmacological inducer of autophagy, was used to increase autophagy in the β-GP-treated VSMCs. 3-methyladenine (3MA) (5 mM), a pharmacological inhibitor of autophagy, was used to decrease autophagy during the osteoblastic differentiation of VSMCs. Treatment with rapamycin led to a significant increase in LC3I/II and Atg5 expression and a robust decrease in Runx2 expression (Fig. 2A), ALP activity (Fig. 2B), Alizarin Red S staining, and calcium deposition (Fig. 2C). Although 3MA attenuated the β-GP-mediated increase in LC3I/II and Atg5 levels, it increased the expression of Runx2 in the 3MA-treated VSMCs (Fig. 2A). This pattern of ALP activity (Fig. 2B) and matrix mineralization (Fig. 2C) is consistent with Runx2 expression; 3MA treatment augmented ALP activity in the cells compared to β-GP treatment alone (Fig. 2B). This trend was also observed for Alizarin Red S staining and calcium deposition (Fig. 2C). To further confirm the pharmacological results of 3MA, siRNA was used to knock down Atg5 expression in VSMCs, thus inhibiting autophagy. We successfully knocked down Atg5 with siRNA #2 (Fig. 2D). Knocking down Atg5 expression significantly augmented β-GP-induced Runx2 expression (Fig. 2E) and ALP activity (Fig. 2F). These data show that increasing autophagy suppresses the osteoblastic differentiation of VSMCs and that inhibiting autophagy has the opposite effect. This is consistent with the findings of a previous study showing that high Pi can promote autophagy in VSMCs and that autophagy plays a protective role by counteracting phosphate-induced vascular calcification^24^.

### Oestrogen inhibits the osteoblastic differentiation of VSMCs *in vitro* and arterial calcification *in vivo*

**Figure 3:**
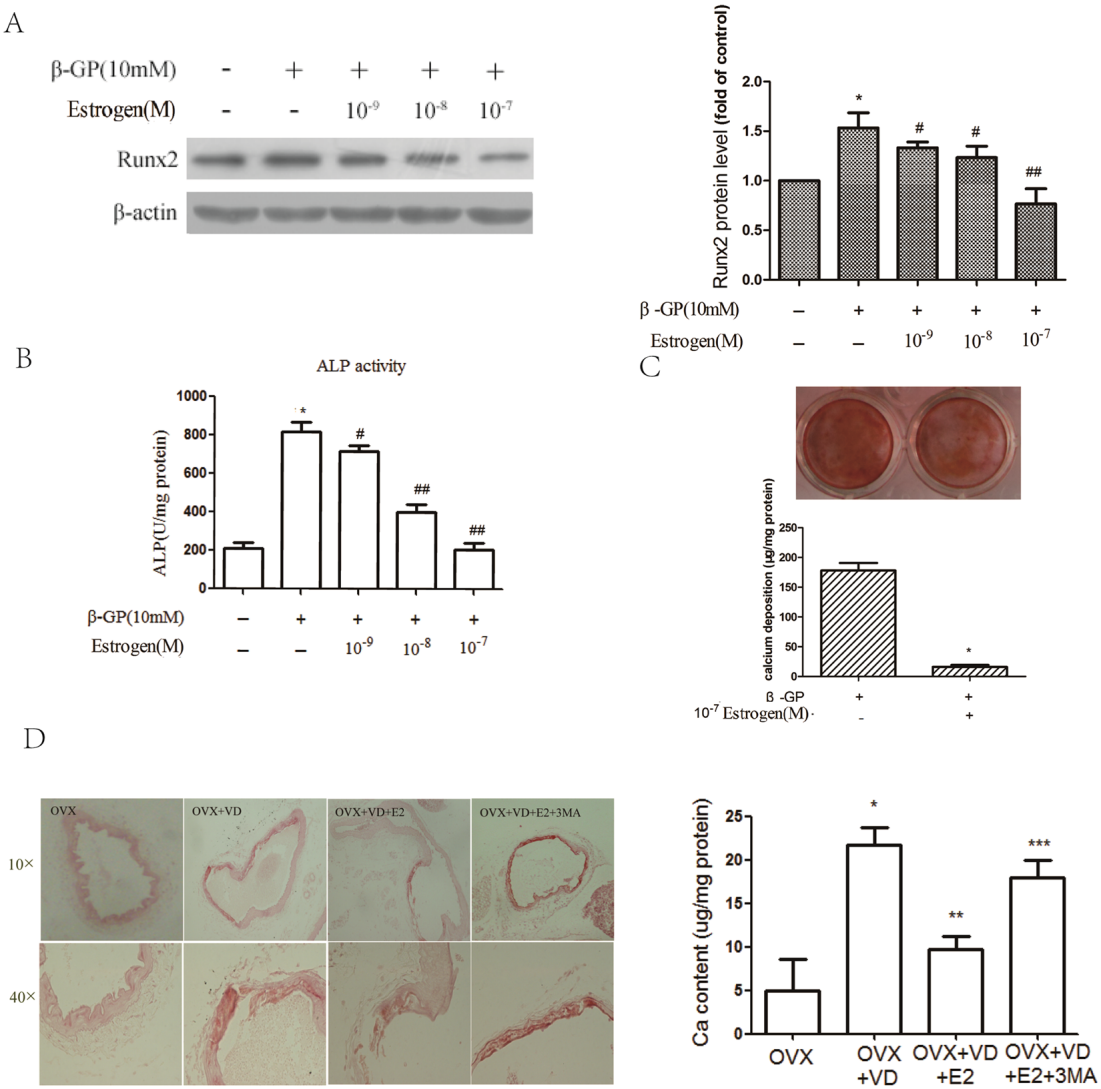
Oestrogen inhibits the calcification of VSMCs *in vitro* and *in vivo*. (**A**,**B**) Analysis of Runx2 levels (**A**) and ALP activity (**B**) in VSMCs treated with different concentrations of oestrogen for 72 h. *p < 0.01 compared with the cells treated with vehicle. ^#^ *p* < 0.05, ^##^ *p *< 0.01 compared with the cells treated with β-GP (n = 5). (**C**) VSMCs were pre-treated with 10^−7^ M oestrogen and β-GP for 12 days. A representative plate view of the Alizarin Red S staining is shown. Quantification of calcium levels using the O-cresolphthalein complexone method. *p < 0.01 compared with the cells treated with vehicle. (**D**) Thirty-two OVX mice were randomly divided into four groups treated with vitamin D3, vitamin D3 + oestrogen, vitamin D3 + oestrogen + 3MA or 7% emulphor (control). Vascular calcification was evaluated by Alizarin Red S staining. (D) OVX mice were randomly divided into four groups and then treated with vitamin D3, vitamin D3 + oestrogen, vitamin D3 + oestrogen + 3MA, or 7% emulphor (control). Calcium content was measured using the O-cresolphthalein complexone method. *p < 0.01 compared with the OVX groups. **p < 0.01 compared with the OVX + VD group. ***p < 0.01 compared with OVX + VD + E2 group (n = 8). Representative images are shown.

To explore the effect of oestrogen on the osteoblastic differentiation of VSMCs, we cultured VSMCs with different concentrations of oestrogen for 72 h. ALP activity and Runx2 expression were determined to identify the effect. As in a previous report showing that oestrogen inhibits vascular calcification^32, 33^, by Western blotting, we demonstrated that treatment with oestrogen significantly inhibited Runx2 expression in a dose-dependent manner. The peak inhibitory effect of oestrogen was observed at a concentration of 10^−7^ M (Fig. 3A). The change in ALP activity and matrix mineralization were similar to that found for Runx2 expression. ALP activity (Fig. 3B), Alizarin Red S staining and calcium deposition (Fig. 3C) were reduced after treatment with oestrogen. In addition, by Alizarin Red staining, we found that oestrogen treatment significantly decreased vitamin D3-induced aortic calcification and calcium content in the arteries of ovariectomized (OVX) mice (Fig. 3D). To investigate whether oestrogen could also regulate the osteoblastic differentiation of VSMCs in OVX mice, we treated VSMCs from OVX mice with oestrogen. By this approach, we found that oestrogen could attenuate the osteoblastic differentiation of VSMCs from OVX or sham-operated mice (Supplemental Figure S1). These data show that oestrogen inhibits the osteoblastic differentiation of VSMCs *in vitro* and arterial calcification and *in vivo*.

### Oestrogen augments the level of autophagy during the osteoblastic differentiation of VSMCs and in calcified arteries

**Figure 4:**
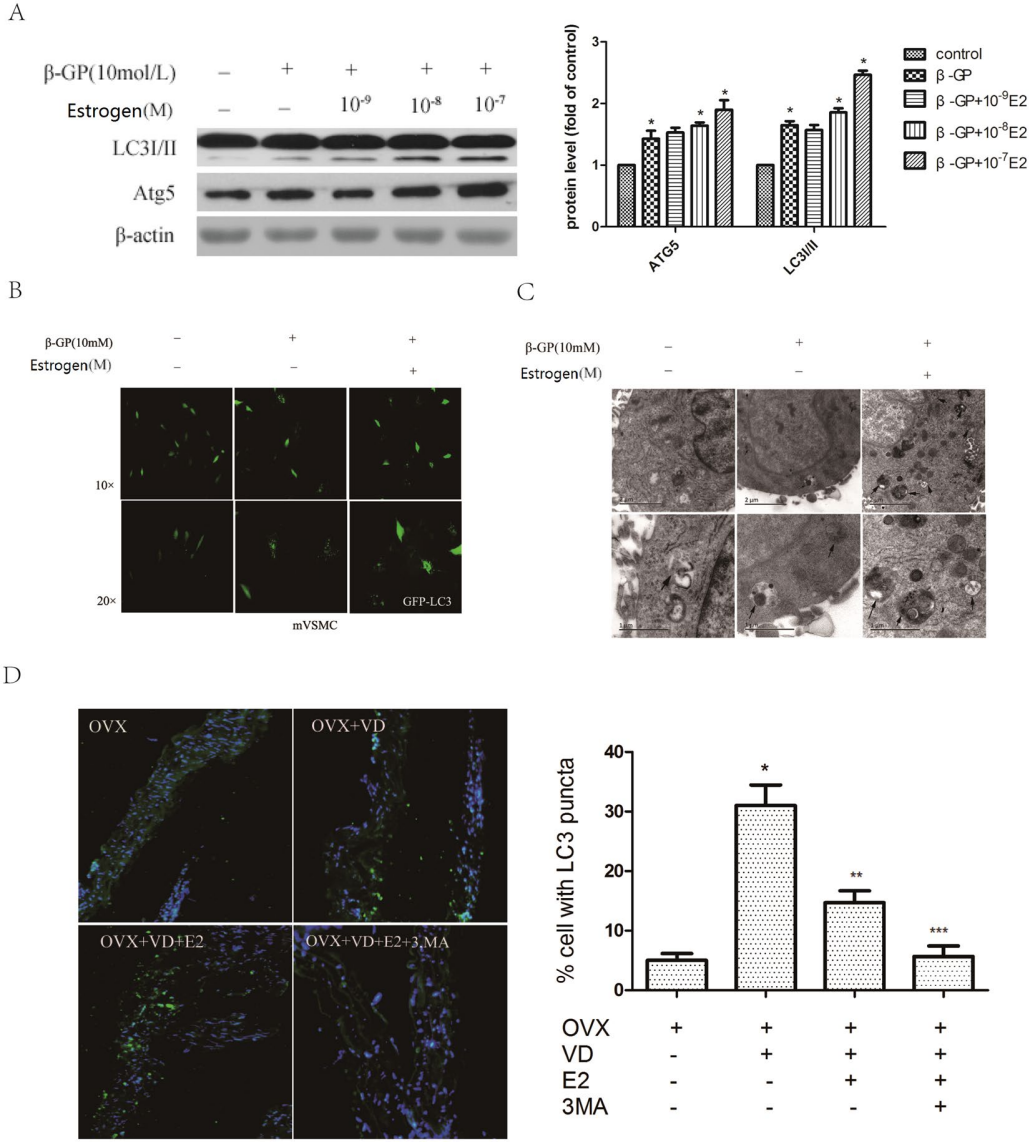
Oestrogen increases the level of autophagy during the osteoblastic differentiation of VSMCs and in calcified arteries. (**A**) Western blot analysis of LC3I/II and Atg5 levels in VSMCs treated with different concentrations of oestrogen for 72 h (n = 5). (**B**) Confocal microscopy of green fluorescent protein (GFP) in VSMCs transiently transfected with GFP-LC3 plasmids cultured for 48 h and then treated with oestrogen for 72 h; autophagosomes are indicated by fluorescent puncta. (**C**) VSMCs were incubated with oestrogen for 72 h and then analysed by electron microscopy. A representative image is shown. The autophagic vacuoles containing organelle remnants are highlighted by arrows (n = 5). (**D**) Immunofluorescence analysis of LC3 puncta in the aorta from four mice subjected to different treatments: OVX mice treated with vitamin D3, vitamin D3 + oestrogen, vitamin D3 + oestrogen + 3MA or 7% emulphor (control) (n = 8). *p < 0.01 compared with the OVX groups. **p < 0.01 compared with OVX + VD group. ***p < 0.01 compared with OVX + VD + E2 group. Representative images are shown.

To determine whether autophagy is involved in the inhibitory effect of oestrogen on vascular calcification, we first examined the effect of oestrogen on autophagosome formation in VSMCs. By Western blotting, we found that oestrogen increased the expression of LC3I/II and Atg5 in a concentration-dependent manner during the osteoblastic differentiation of VSMCs (Fig. 4A). The increased accumulation of LC3 puncta tagged with GFP shows that oestrogen can increase the level of autophagy (Fig. 4B). Electron microscopy of typical autophagic structures provided us with direct evidence to support the increase in autophagy by oestrogen; more autophagosomes were found in the VSMCs treated with oestrogen than in the control cells (Fig. 4C). Immunofluorescence analysis revealed the accumulation of more LC3 puncta in the arteries from mice treated with vitamin D3 plus oestrogen than in those from mice treated with vitamin D alone (Fig. 4D). Taken together, these data demonstrate that oestrogen further augments the level of autophagy during the osteoblastic differentiation of VSMCs and in calcified arteries.

### Oestrogen inhibits the osteoblastic differentiation of VSMCs by promoting autophagy

**Figure 5:**
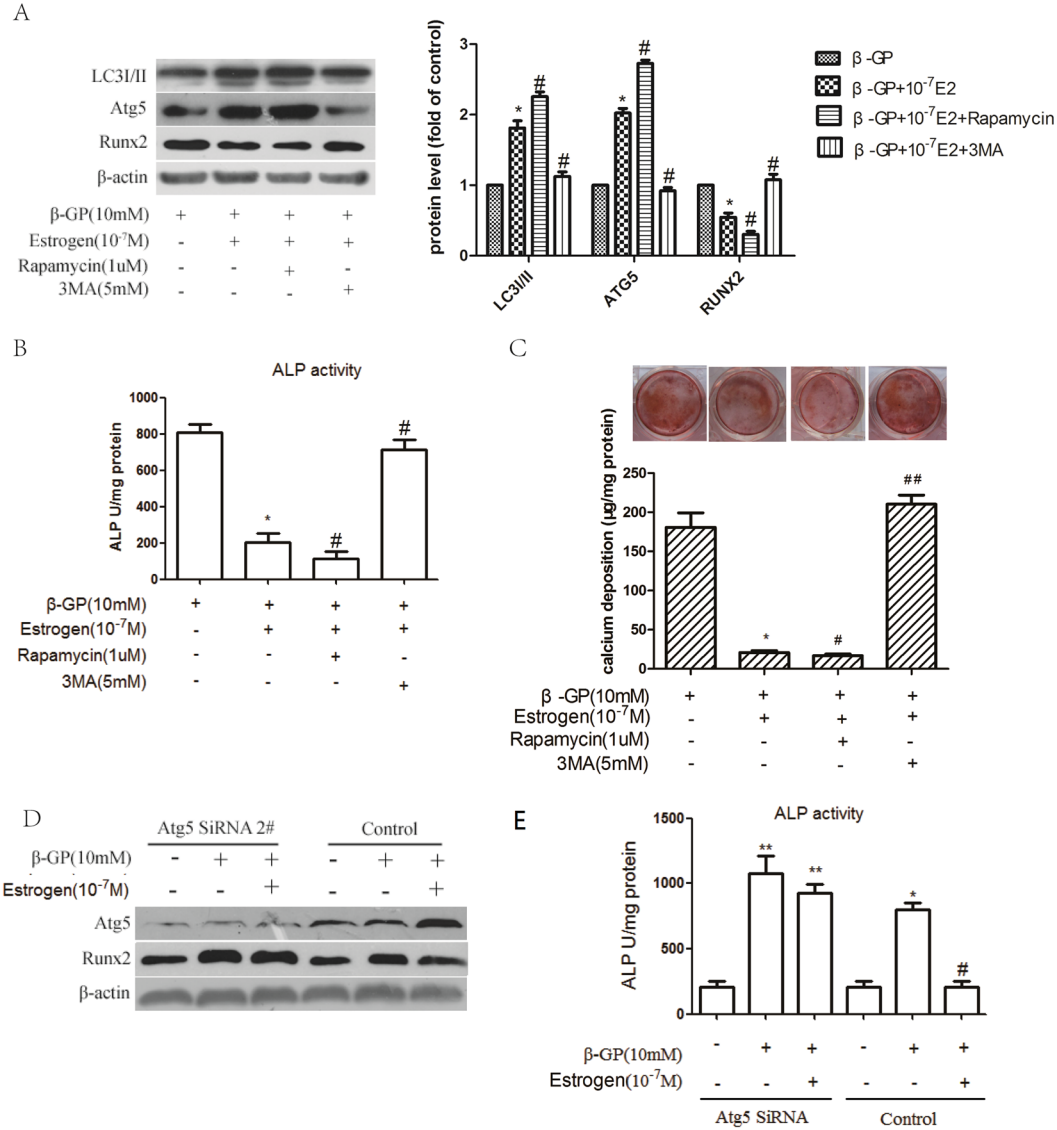
Oestrogen inhibits the calcification of VSMCs via promotion of autophagy. (**A**,**B**) VSMCs were pre-treated with the indicated concentrations of 3MA or rapamycin for 30 min and subsequently incubated with medium containing β-GP and oestrogen for 72 h. LC3I/II, Atg5 and Runx2 levels (**A**) and ALP activity (**B**) were detected using Western blot analysis or an ALP kit, respectively. *p < 0.01 compared with the cells treated with β-GP. ^#^ *p* < 0.01 compared with the cells treated with β-GP and oestrogen (n = 5). (**C**) VSMCs were pre-treated with the indicated concentrations of 3MA or rapamycin for 30 min and then incubated with β-GP and oestrogen for 12 days. A representative plate view of the Alizarin Red S staining is shown. Quantification of calcium levels using the O-cresolphthalein complexone method. *p < 0.01 compared with the cells treated with β-GP. ^#^ *p* < 0.05 and ^##^ *p* < 0.01 compared with the cells treated with β-GP and oestrogen (n = 5). (**D**,**E**) VSMCs were transfected with control or Atg5 2# siRNA for 48 h and treated with or without β-GP and oestrogen for 72 h. Western blot analysis of Atg5 and Runx2 protein levels (**D**) and ALP activity detection (**E**) were performed. *p < 0.01 compared with the cells treated with vehicle in the control group and ^#^ *p* < 0.01 compared with the cells treated with β-GP in the control group. **p < 0.01 compared with the cells treated with vehicle in the ATG5 siRNA group (n = 5). Representative images are shown.

Because the oestrogen-treated cells showed higher levels of autophagy and oestrogen inhibited the osteoblastic differentiation of VSMCs, we next determined whether autophagy was involved in the inhibition of vascular calcification by oestrogen. Treatment with rapamycin slightly increased the level of autophagy induced by oestrogen, demonstrated by the upregulation of LC3I/II and Atg5 expression (Fig. 5A). In the presence of rapamycin, Runx2 expression, ALP activity and Alizarin Red S staining were further decreased (Fig. 5A–C). Compared with treatment with oestrogen alone, treatment with 3MA significantly inhibited oestrogen-induced autophagy, reflected by the dramatic decrease in LC3I/II and Atg5 expression (Fig. 5A), whereas Runx2 expression, ALP activity and Alizarin Red S staining and calcium deposition were significantly increased (Fig. 5A–C). Similarly, the siRNA-mediated knockdown of Atg5 resulted in a significant augmentation of Runx2 expression and ALP activity (Fig. 5D and E). As noted previously, oestrogen could significantly alleviate the osteoblastic differentiation of VSMCs, and rapamycin augmented the inhibitory effect of oestrogen, and the inhibitory effect of oestrogen could be counteracted by 3MA or knockdown of Atg5. In the mouse model of arterial calcification, oestrogen attenuated the vascular calcification induced by vitamin D3 in OVX mice. Vascular calcification was increased significantly in the 3MA plus oestrogen-treated group compared with that in the oestrogen-treated group (Fig. 3D). Autophagy was decreased in the 3MA-treated group, which was reflected by the dramatic decrease in the accumulation of LC3 puncta (Fig. 4D). Collectively, these results indicate that oestrogen inhibits the osteoblastic differentiation of VSMCs and arterial calcification via the promotion of autophagy. Rapamycin enhanced the inhibitory effect of oestrogen on the osteoblastic differentiation of VSMCs. Thus, both *in vitro* and *in vivo*, the inhibitory effect of oestrogen on calcification was attenuated by blocking oestrogen-induced autophagy.

### The inhibitory effect of oestrogen on VSMCs involves an increase in autophagy mediated by ERα but not ERβ

**Figure 6:**
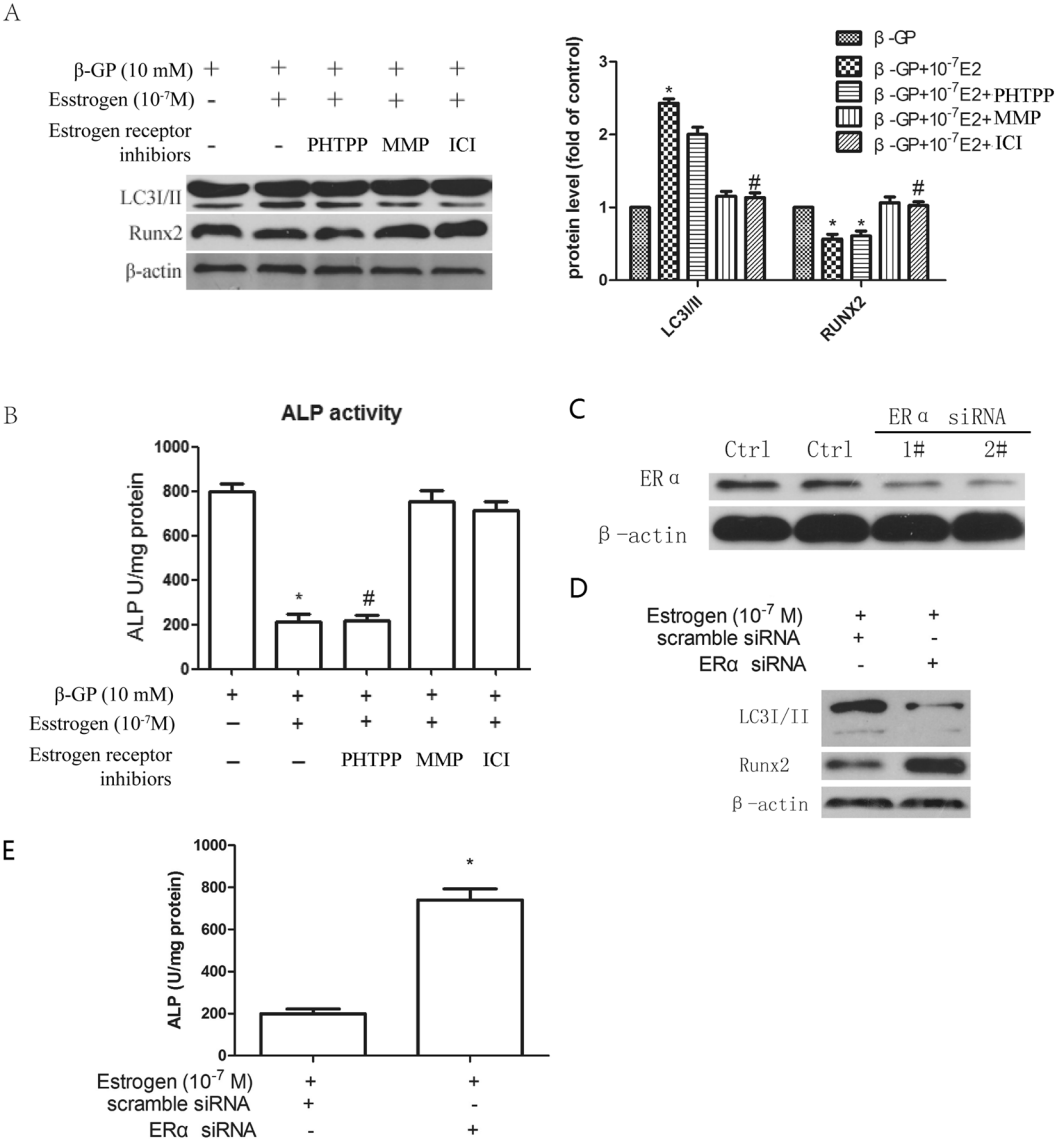
Autophagy is induced by oestrogen via ERα but not ERβ. (**A**) VSMCs were incubated with medium containing β-GP and then pre-treated with ICI 182780 (100 nM, an ER antagonist), MPP (100 nM, a selective ERα antagonist) or PHTPP (100 nM, a selective ERβ antagonist) for 30 min and subsequently incubated with oestrogen for 72 h. LC3I/II and Runx2 levels (**A**) and ALP activity (**B**) were detected using Western blot analysis or an ALP kit, respectively. *p < 0.01 compared with the cells treated with β-GP. ^#^ *p* < 0.01 compared with the cells treated with β-GP + oestrogen group (n = 5). (**C**–**E**) VSMCs were transfected with scrambled control or ER-specific siRNA for 48 h and then treated with β-GP and oestrogen for 72 h. Western blot analysis of Atg5 and Runx2 protein levels (**D**) and ALP activity detection (**E**) were performed. *p < 0.05 compared with the cells treated with oestrogen and scrambled control. Representative images are shown.

It is now widely accepted that the influence of oestrogen on target tissues is primarily mediated by two oestrogen receptors (ERs), oestrogen receptor alpha (ERα) and oestrogen receptor beta (ERβ). ERs play crucial roles in proliferation, differentiation, migration and apoptosis by modulating distinct genomic and/or nongenomic pathways. To investigate whether the inhibitory effect of oestrogen on calcification is related to ER isoforms, the effects of two specific ER isoform antagonists (MPP for ERα and PHTPP for ERβ) and fulvestrant (ICI 182780) (antagonist for both ERα and ERβ) on the osteoblastic differentiation of VSMCs were investigated. MPP and ICI 182780 counteracted the oestrogen-induced changes in Runx2 expression and ALP activity (Fig. 6A and B). In contrast, the administration of PHTPP had no obvious effect on Runx2 expression and ALP activity in the oestrogen-treated VSMCs (Fig. 6A and B). Furthermore, when the oestrogen-treated cells were treated with MPP or ICI 182780, the expression of LC3I/II was decreased. This effect was not observed when the cells were treated with PHTPP (Fig. 6A). Moreover, to verify that ERα mediated the effect of oestrogen on the osteoblastic differentiation of VSMCs, we knocked down ERα expression using ERα-specific siRNA (Fig. 6C). We found that the ERα-specific siRNA attenuated the repressive effects of oestrogen on Runx2 expression and ALP activity (Fig. 6D and E). Additionally, we found that the oestrogen-induced expression of LC3I/II was blocked by the ERα-specific siRNA (Fig. 6D). These results show that the inhibitory effect of oestrogen on the osteoblastic differentiation of VSMCs involves the promotion of autophagy mediated by ERα but not ERβ.

## Discussion

Here, using *in vitro* and *in vivo* mouse models of arterial calcification, we found that autophagy plays a vital endogenous protective role during the osteoblastic differentiation of VSMCs. Moreover, oestrogen directly potentiated autophagy, which attenuated the osteoblastic differentiation of VSMCs *in vitro* and arterial calcification *in vivo*. The inhibition of autophagy by 3MA treatment or siRNA-mediated knockdown of the autophagy protein Atg5 significantly ameliorated the inhibitory effect of oestrogen on the osteoblastic differentiation of VSMCs. Rapamycin, a pharmacological inducer of autophagy, significantly enhanced the protective effect of oestrogen. Additionally, oestrogen attenuated arterial calcification through the inhibition of autophagy in a mouse model of arterial calcification, as demonstrated by the blockage of the effect of oestrogen by 3MA. Blocking ERα (either using an ERα inhibitor or a specific siRNA) attenuated the effect of oestrogen on the osteoblastic differentiation of VSMCs. Thus, targeting the autophagic pathway may help to prevent or treat vascular calcification, and this provides a theoretical basis for oestrogen in the attenuation of vascular calcification.

Autophagy is a multifunctional process involved in various cellular activities and is essential for survival, differentiation and development. By manipulating autophagy using several experimental procedures, multiple lines of evidence have shown that autophagy plays a vital role in neurodegenerative diseases, cancer and cardiovascular diseases, thereby representing a potential target in the development of therapeutic strategies in these diseases^12^. A previous study also reported far-reaching roles of autophagy in osteoporosis, and the inhibition of autophagy was shown to lead to osteopenia in mice through the inhibition of osteoblast differentiation^34^. Regarding vascular calcification, a more recent study has shown that induction of autophagy by atorvastatin suppressed the TGF-β1-stimulated calcification of VSMCs^35^. Autophagy plays an endogenous protective role in vascular calcification in the context of hyperphosphatemia^24^. To test whether autophagy is directly associated with the calcification of VSMCs, we investigated the effect of autophagy in the process of β-GP-induced calcification of VSMCs. First, we demonstrated that β-GP induced autophagy during the osteoblastic differentiation of VSMCs based on the elevation of LC3I/II and Atg5 expression (by Western blotting), stimulation of GFP–LC3 redistribution (by immunofluorescence analysis) and the accumulation of typical autophagic structures (by electron microscopy). Moreover, immunofluorescence analysis confirmed the formation of autophagosomes in the renal arteries from uremic patients but not in those from healthy donors. These phenomena demonstrate that autophagy plays an important role in arterial calcification. Next, we investigated the function of autophagy in the calcification of VSMCs. The inhibition of autophagy by 3MA or ATG5-knockdown promoted the osteogenic differentiation of VSMCs, indicated by increased ALP activity and Runx2 protein expression. In contrast, the promotion of autophagy by rapamycin attenuated the osteogenic differentiation of VSMCs. These results indicate that autophagy inhibits the osteogenic differentiation of VSMCs *in vitro*.

Arterial calcification is an actively regulated process that is similar to osteogenesis. The process of transdifferentiation of VSMCs to the osteogenic phenotype plays a crucial role in arterial calcification^36^. Epidemiological studies have shown that the incidence of aortic calcification is intimately related to low bone mineral density in postmenopausal women; this phenomenon is known as the calcification paradox^37, 38^. These studies suggest that oestrogen plays an important role in the development of arterial calcification. Our data show that the activity of ALP, a well-recognized early marker of osteoblastic differentiation, is increased by treatment with β-GP and that oestrogen can reduce ALP activity during the osteoblastic differentiation of VSMCs. We also show that oestrogen reduces the expression of Runx2 (an important transcription factor in osteoblast differentiation) during the osteoblastic differentiation of VSMCs. Additionally, Alizarin Red S staining showed that oestrogen attenuates vascular calcification *in vitro* and *in vivo*. These results demonstrate that oestrogen can inhibit arterial calcification *in vitro* and *in vivo*.

A previous study has shown that oestradiol-induced autophagy plays a protective role in the survival of osteoblasts^39^. A contradictory association that vascular calcification is frequently accompanied by low bone mineral density or disturbed bone turnover has been widely reported^38^. Our study confirmed that autophagy functionally contributes to the inhibitory effect of oestrogen on vascular calcification. We used three different methods to verify that oestrogen can induce autophagy in VSMCs *in vitro*: 1) Western blotting, 2) analysis of autophagy by GFP–LC3 redistribution and 3) electron microscopy. Immunofluorescence analysis further demonstrated that oestrogen induced autophagy in a mouse model of arterial calcification. The relationship between the osteogenic differentiation of VSMCs and oestrogen-induced autophagy is supported by our findings that the inhibition of autophagy by the pharmacological inhibitor 3MA or by siRNA-mediated knockdown of Atg5 significantly alleviated the protective effect of oestrogen on the calcification of VSMCs, indicating that autophagy is a target through which oestrogen inhibits VSMC calcification. Moreover, as expected, the pharmacological inducer of autophagy rapamycin demonstrated a cumulative inhibitory effect on the calcification of VSMCs that were exposed to oestrogen. We also showed that 3MA attenuates the inhibitory effect of oestrogen on aortic calcification in a mouse model of arterial calcification. Thus, our findings are in line with the calcification paradox, as previous studies have shown that autophagy favours the osteogenic differentiation of mesenchymal stem cells^40^ and that oestrogen-induced autophagy plays a protective role in osteoblast survival^39^. Our findings demonstrate that autophagy stimulated by oestrogen plays a protective role in vascular calcification both *in vitro* and *in vivo*.

It is now widely accepted that ERs play crucial roles in proliferation, differentiation, migration and apoptosis by modulating distinct genomic and/or nongenomic pathways. The role of ERα in inhibiting VSMC proliferation has been well established for decades; our findings show that oestrogen-promoted autophagy was blocked by ICI 182780 (an oestrogen receptor antagonist), MPP (a selective ERα receptor antagonist) or ERα-specific siRNA but not by PHTPP (a selective ERβ receptor antagonist). These results indicate that autophagy is induced by the binding of oestrogen to ERα but not to ERβ.

However, a large preventive trial in the United States failed to confirm the protective effect of oestrogen alone or oestrogen combined with progestin hormone therapy on coronary heart disease. Subgroup analyses have shown that the women who were given hormone therapy beginning at a younger age (50–59 years) or earlier after menopause tended to have a reduced risk of coronary heart disease and total mortality^25^. An ancillary randomized study found a significant reduction in the coronary artery calcium scores among younger (50–59 years) women who received conjugated oestrogen compared with the women who received a placebo, indicating a reduced burden of calcified plaques^41^. Our study provides a theoretical foundation for epidemiological studies showing that oestrogen can inhibit the calcification of VSMCs by regulating autophagy.

Several epidemiological studies have suggested a relationship between vascular calcification and impaired bone metabolism, especially in postmenopausal women. Oestrogen replacement therapy has been shown to be effective for improving bone mineral density in osteoporotic postmenopausal women. Basal autophagy plays an important role in regulating terminal differentiation and bone growth, as well as in maintaining osteoblast/osteocyte survival. The findings presented here show that autophagy induced by oestrogen is a protective mechanism counteracting vascular calcification via the ERα signalling pathway. This provides a theoretical foundation for the use of oestrogen in the treatment of ageing- or disease-related vascular calcification and osteoporosis in women.

**Figure 7:**
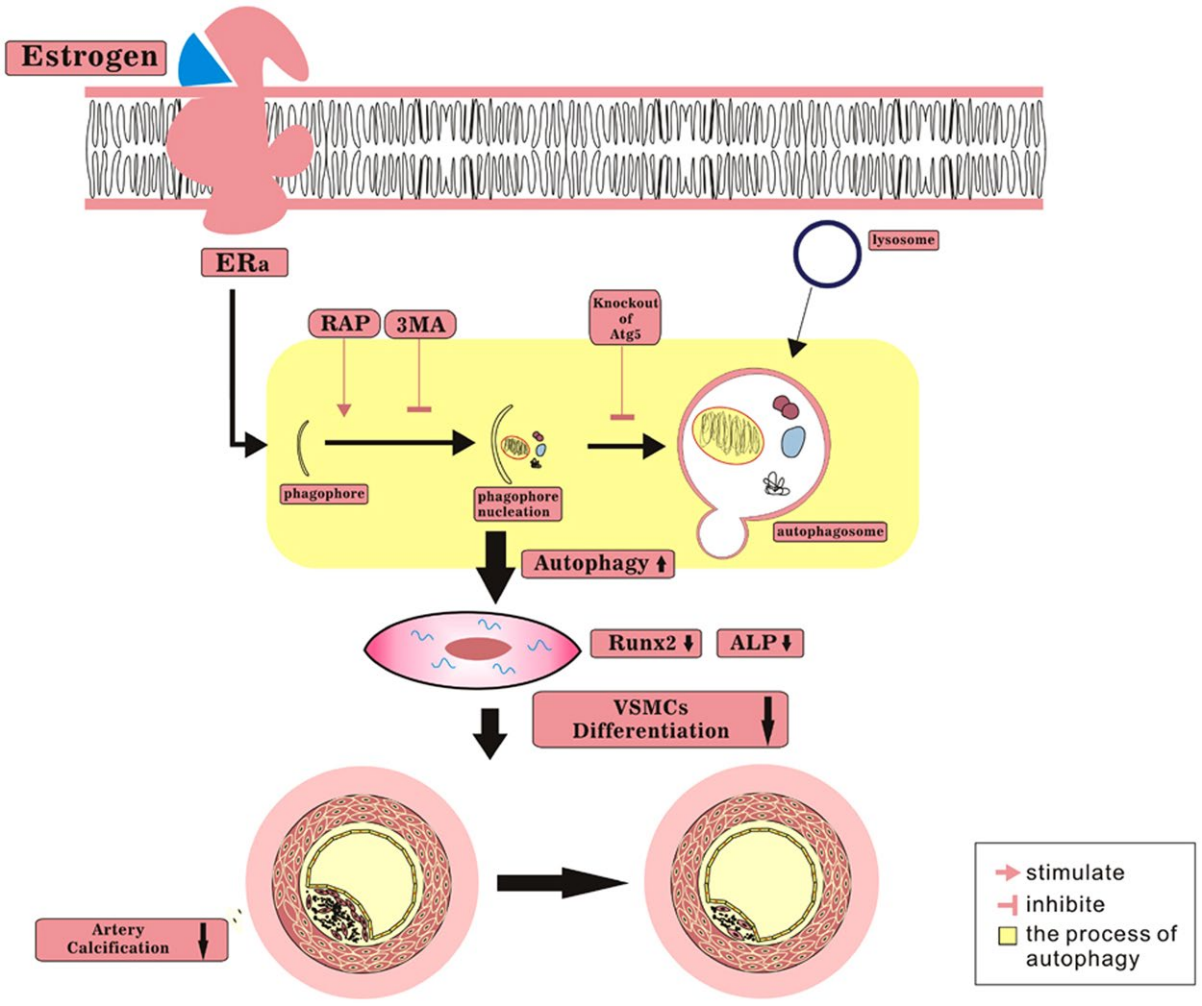
A proposed model of the effect of oestrogen on arterial calcification. Oestrogen enhanced the level of autophagy, which inhibited the differentiation of VSMCs and arterial calcification through ERα. Rapamycin further enhanced the inhibitory effect of oestrogen, whereas 3MA or knockdown of Atg5 impaired the inhibitory effect of oestrogen.

In conclusion, this study found that oestrogen-induced autophagy plays a protective role in VSMCs *in vitro* and inhibits medial artery calcification *in vivo*, which represents a novel mechanism for the regulation of vascular calcification. As depicted in Fig. 7, oestrogen-induced autophagy inhibits the osteogenic differentiation of VSMCs and arterial calcification via the ERα pathway. This finding is relevant to the prevention and treatment of a variety of cardiovascular diseases related to vascular calcification. The investigation of autophagy in VSMCs will provide us with a better understanding of the mechanisms and promote the development of autophagy-related drugs designed for the treatment of cardiovascular diseases. Moreover, our results provide evidence for a protective role of oestrogen against the calcification of VSMCs and further elucidate the signalling mechanisms that can potentially be exploited in the treatment of vascular calcification-related cardiovascular diseases.

## Materials and Methods

### Ethics statement

The animal and clinical studies were approved by the Ethics Committee of the Second Xiangya Hospital of Central South University. The clinical study conformed to the principles of the Declaration of Helsinki and written informed consent was obtained from all participants. The animal investigation conformed to the Guide for the Care and Use of Laboratory Animals, US National Research Council-2011.

### Reagents and antibodies

β-GP (50020), 17-β estrogen (E8875), Rapamycin (37094), 3MA (M9281) and Fulvestrant (V900926) were from Sigma-Aldrich. Methy-piperidinopyrazole (MPP, ERα selective antagonist) and 2-phenyl-3-(4-hydroxyphenyl)-5, 7-bis (trifluoromethyl)-pyrazolo [1,5-alpha] pyrimidine (PHTPP, ERβ selective antagonist) were from Tocris, and anti-LC3I/II was from Cell Signaling. Anti-β-actin was from Santa Cruz Biotechnology, and anti-Runx2 and anti-ATG5 were from Abgent. The GFP–LC3 expression vector was from Origene and Lipofectamine 2000 was from Invitrogen. siRNA against mouse Atg5 was synthesized by GeneChem. The ALP kit was from Jiancheng Nanjing Biological Engineering, and Alexa Fluor R488 donkey anti-rabbit was from Abcam. Dulbecco’s Modified Eagle’s medium (DMEM) and foetal bovine serum (FBS) were from Gibco-BRL, and RIPA lysate was from Beyotime.

### Cell culture

Mouse vascular smooth muscle cells (mVSMCs) were acquired from 8-week-old female C57/BL6 OVX mice or mice with intact ovaries. Briefly, the mice were sacrificed by CO_2_ inhalation/cervical dislocation, the thoracic aorta was carefully dissected out, and the tunica media was isolated from the mouse aorta. After the adventitia was removed, the tissue was fragmented (1–2 mm^3^), and the aortas were minced and digested in 5 ml of digestion solution (0.125 mg/ml of elastase, 0.25 mg/ml of soybean trypsin inhibitor, 10 mg/ml of collagenase I, 2.0 mg/ml of crystallized bovine albumin and 15 mM HEPES) at 37 °C for 45 min. The cellular digests were filtered through sterile 100-mM nylon mesh, centrifuged at 1000 rpm for 10 min and washed twice in DMEM containing 4.5 g/L of glucose and 10% FBS (Gibco-BRL Corp, NY, USA) before culturing in the same medium. mVSMCs were isolated from the same batch of cells, the experiments were performed between passages three and eight from the primary culture, and the extra cells were stored in liquid nitrogen. Immunocytochemical examination showed positive staining in all cells for α-smooth muscle actin. mVSMCs were cultured in DMEM containing 4.5 g/L of glucose, 10% FBS and 10 mM sodium pyruvate, and the medium was refreshed every 2–3 days. mVSMCs were cultured in medium containing 10 mM β-GP (Sigma-Aldrich, USA) to induce the osteoblastic differentiation of VSMCs. To reveal the effect of oestrogen on the osteoblastic differentiation of VSMCs and the mechanism involved, we incubated VSMCs with 10^−9^ to 10^−7^ M of 17-β oestrogen (Sigma-Aldrich) in the subsequent experiments. To investigate the effect of autophagy on the calcification of VSMCs, cells were pre-treated with the autophagy inhibitor 3MA (Sigma-Aldrich, 5 mM) or the autophagy inducer rapamycin (RAP, Sigma-Aldrich, 10 µM) for 30 min.

### Western blot analysis

Cell extracts were collected after treatment for the indicated times. The total protein extracts of cultured cells were prepared with RIPA lysate (Beyotime, China). Equal amounts of protein were subjected to SDS-PAGE and transferred onto PVDF membranes (Pall, USA). Then, the membranes were incubated successively with 5% non-fat milk and stained with primary antibodies anti-LC3 (1:500, CST, Danvers, MA), anti-β-actin (1:1000, Santa Cruz Biotechnology, Santa Cruz, CA), anti-Runx2 (1:500, Abgent, San Diego, CA) or anti-ATG5 (1:500, Abgent, San Diego, CA). The membranes were then incubated with goat anti-mouse or anti-rabbit IgG antibody conjugated with horseradish peroxidase in 2% milk for 1 h. Finally, the reaction was visualized by chemiluminescence^42–44^.

### Analysis of autophagy by GFP–LC3 redistribution

After plating for 24 h, cells were transfected with GFP–LC3 expression vector (Origene, USA) using Lipofectamine 2000 (Invitrogen, Grand Island, NY). Next, 6 h later, the transfection medium was replaced with DMEM containing 10% FBS. The next day, cells were treated with β-GP and/or estrogen for 72 h. The GFP signal was monitored by confocal laser scanning microscopy (Leica, Bannockburn, IL). The following criteria were used to identify cells with punctuating GFP–LC3 (positive cells): (1) uneven, ring-shaped dots in the cytoplasm; (2) more dots than the mean number of normal cells.

### RNA interference

RNA interference was used to silence the expression of Atg5 and ERα in VSMCs. siRNA against mouse Atg5 or ERα and their respective scrambled controls were synthesized by GeneChem (Shanghai, China). VSMCs were cultured in 6-well plates for 24 h in medium without antibiotics. The cells were transfected with siRNAs at a final concentration of 100 pmol using Lipofectamine 2000 (Invitrogen, USA) according to the manufacturer’s instructions and then incubated for 6 h before the addition of 10% FBS for 48 h. At the end of treatment, the cells were harvested for experiments. The efficiency of gene knockdown was confirmed by Western blot analysis.

### Electron microscopy

Electron microscopy was performed at the Department of Medical Ultrastructure, School of Basic Medicine, Laboratory of Biomedical Electronic Microscopy of Higher Research Center, Central South University. Briefly, cells were pre-fixed with 2.5% glutaraldehyde and post-fixed with 1% osmium tetroxide. After dehydration, the cells were embedded in epoxy resin that had been solidified in an oven. Ultrathin sections were cut, stained with uranyl acetate and lead nitrate, and examined under a transmission electron microscope (FEI, Hillsboro, USA).

### Measurement of ALP activity, mineralized matrix formation and calcium content

Cells were cultured for the indicated times and subjected to different treatments, and then the cells were washed three times with phosphate-buffered saline (PBS). The cells were homogenized with a solution containing 20 mM Tris-HCl, pH 8.0, 150 mM NaCl, 1% Triton X-100, 0.02% NaN_3_ and 1 mM PMSF and centrifuged in a microfuge at 12,000 *g* for 10 min. The supernatant was removed for ALP and protein concentration assays. ALP activity was measured colourimetrically from the hydrolysis of p-nitrophenyl Pi using an ALP kit. The results were normalized to the levels of total protein.

To measure the formation of mineralized matrix, Alizarin Red S staining was performed. Briefly, VSMCs were fixed in 70% ethanol at room temperature for 1 h. The cells were then stained with 40 mM Alizarin Red S for 10 min. Next, the cells were washed three times with PBS to remove nonspecific staining.

For the quantification of calcium levels, the cells or dried artery samples were decalcified with HCl. The calcium content in HCl supernatants was determined using the O-cresolphthalein complexone method. Total protein was quantified using the Bradford protein assay. The calcium content was normalized to the protein content and expressed as micrograms calcium per milligram protein.

### Animal experiments

Thirty-two 6-week-old female C57/BL6 mice were ovariectomized under anaesthesia (by Nembutal 40 mg/kg i.m.). Two weeks later, the mice were randomly divided in four groups: vitamin D3 + vehicle (n = 8), vitamin D3 + oestrogen (n = 8), vitamin D3 + oestrogen + 3MA (n = 8), and control (n = 8). The mice in the vitamin D3 + vehicle group were given intraperitoneal injections of vitamin D3 at a dose of 500 000 IU/kg body weight on days 1–4 and vehicle on days 1–14 to induce arterial calcification. The mice in the vitamin D3 + oestrogen group received vitamin D3 on days 1–4 and 20 µg/kg body weight of oestrogen on days 1–14 by intraperitoneal injection. The mice in the vitamin D3 + oestrogen + 3MA group received vitamin D3 on days 1–4, 20 µg/kg body weight of oestrogen and 100 mg/kg body weight of 3MA on days 1–14 by intraperitoneal injection. The mice in the control group received intraperitoneal injections of 7% emulphor (vitamin D3 stock solution) on days 1–4 and vehicle on days 1–14. The mice were sacrificed via CO_2_ inhalation/cervical dislocation 2 weeks after the first injection. Arteries were dissected from the mice and fixed in 4% paraformaldehyde for 24 h and then embedded in paraffin. Alizarin Red S staining was used to detect medial artery calcification. Dried artery samples were deparaffinised with turpentine oil. After three washes with PBS, the arteries were stained with to 1% Alizarin Red S for 10 min and washed with PBS. The positively stained cells showed a reddish colour.

### Patients and arterial tissue samples

Renal arterial samples from a total of 10 pairs of uremic patients scheduled to undergo kidney transplantation and from healthy donors were obtained from the Center of Organ Transplantation, the Second Xiangya Hospital of Central South University. Written informed consent was obtained from all patients in this study.

### Immunofluorescence analysis

After deparaffinisation and antigen retrieval, the artery samples were permeabilised by incubation in 0.1% Triton X-100 in 5% bovine serum albumin–PBS for 15 min; the samples were then treated with anti-LC3 antibody (1:200) overnight at 4 °C, followed by treatment with secondary antibody (Alexa Fluor 488 donkey anti-rabbit IgG, 1:100) for 1 h at 37 °C. The nuclei were stained with DAPI. The primary antibody was replaced with normal IgG for the negative control.

### Statistical analysis

The results of the experiments are presented as means ± standard deviation (SD), and analysis was performed with Statistical Product and Service Solutions (SPSS) software (version 17.0). Comparisons between values of more than two groups were evaluated by one-way ANOVA. A level of *p* < 0.05 was considered statistically significant. Representative experiments are shown in the figures.

## Electronic supplementary material

Supplementary material
