# Supplementary material for: Oestrogen Inhibits Arterial Calcification by Promoting Autophagy
Source: Sci Rep. 2017 Jun 14;7:3549. doi: 10.1038/s41598-017-03801-x (PMC5471178; doi:10.1038/s41598-017-03801-x)
Supplement: Supplementary file 1 — Supplementary material [file 41598_2017_3801_MOESM1_ESM.doc]

# Title: Estrogen Inhibits Arterial Calcification by Promoting Autophagy

**Yi-Qun Peng^1^, *, Dan Xiong^1, 2^ *, Xiao Lin^1^, Rong-Rong Cui^1^, Feng Xu^1^, Jia-Yu Zhong^1^, Ting Zhu^1^, Feng Wu^3^, Min-Zhi Mao^4^, Xiao-Bo Liao^5^, Ling-Qing Yuan^1, #^**

1. Department of Metabolism and Endocrinology, National Clinical Research Center for Metabolic Diseases, The Second Xiang-Ya Hospital, Central South University, Changsha, Hunan, People’s Republic of China
2. Department of Endocrinology, Central hospital of Yiyang, Yiyuang, Hunan, People’s Republic of China
3. Department of Pathology, The Second Xiang-Ya Hospital, Central South University, Changsha, Hunan, People’s Republic of China
4. Department of Orthopaedics, The Second Xiang-Ya Hospital, Central South University, Changsha, Hunan, People’s Republic of China
5. Departments of Cardiothoracic Surgery, The Second Xiang-Ya Hospital, Central South University, Changsha, Hunan, People’s Republic of China

***** These authors equally contributed to this work.

**#Corresponding author:** Ling-Qing Yuan, Department of Metabolism and Endocrinology, The Second Xiangya Hospital, Central South University, Changsha, Hunan, 410011, China. Fax: 86731-85361472 Telephone: 8613077371004, E-mail address: allenylq@hotmail.com

Table 1 characterristics of donors and recipients for kidney transplation

|  | Donors (n=10) | Recipients(CKD patients n=10) |
| --- | --- | --- |
| Age | 46±10(19-65) | 32±9(19-62) |
| Males sex n(%) | 7(70%) | 7(70%) |
| Serum crentimine (umol/L) | 60.2±12.4 | 987.2±216.1 |
| eGFR (ml/min /1.73m^2^) | 115.15±19.21 | 48.31±29.34 |
| Cause of CKD (n) |  |  |
| Glomerulonephritis |  | 7 |
| Diabetic nephropathy |  | 1 |
| others |  | 2 |

A


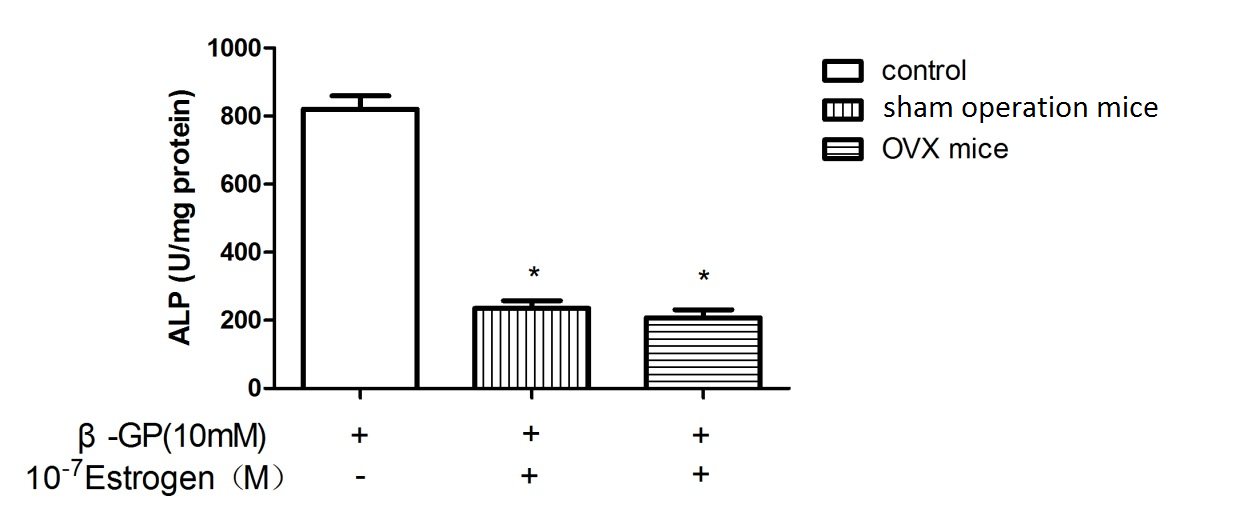


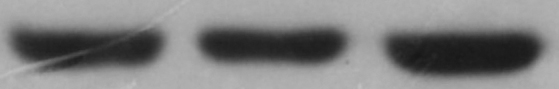

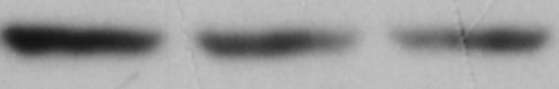


Runx2

β-actin

B

β-GP (10mM) + + +

10^-7^ Estrogen (M) - + +

Supplemental Figure 1: VSMCs from sham operation mice and OVX mice were cultured with β-GP, and then treated with estrogen. (A) ALP activity was measured by using an ALP kit, (B) Runx2 levels was determined using western blotting. **p＜0.05*
